# Anatomical study of the coracoid process in Mongolian male cadavers using the Latarjet procedure

**DOI:** 10.1186/s13018-016-0461-3

**Published:** 2016-10-24

**Authors:** Jianqiang Lian, Lele Dong, Yanjun Zhao, Jinlei Sun, Wenlong Zhang, Chunzheng Gao

**Affiliations:** 1 The First Affiliated Hospital of Baotou Medical College, Baotou, Inner Mongolia China; 2 The Second Hospital of Shandong University, Jinan, Shandong China

**Keywords:** Shoulder, Coracoid, Anatomy, Arthroscopy, Latarjet

## Abstract

**Background:**

The Latarjet procedure addresses recurrent anterior shoulder instability in the context of a significant bony defect. However, the bony and soft tissue anatomy of the coracoid in coracoid transfer procedures has not yet been defined in Mongolian men. The aims of this study were to describe the soft tissue attachments of the coracoid regarding the bony anatomy, define the average amount of bone available for coracoid transfer, analyze the characteristics of the pectoralis minor and coracoid, and study the relationship between the bony dimensions of the coracoid and body length in Mongolian men.

**Methods:**

We dissected 30 shoulders from 15 male Mongolian cadavers, exposing the coracoid process and attached anatomical structures including the lateral clavicle and acromion, then measured the bony dimensions of the coracoid and the locations and sizes of the coracoid soft tissue footprints.

**Results:**

The mean length of the coracoid available for transfer was 23.93 ± 2.32 mm. The mean length of the coracoid was 42.10 ± 2.3 mm, and the mean width and height of the coracoid midpoint were 15.29 ± 1.70 mm and 11.61 ± 1.98 mm, respectively. The pectoralis minor was part of the joint capsule and passed over the coracoid in some samples. The mutation rate of the pectoralis minor footprint, which was asymmetrical and irregular, was 23.33 %. Statistical analysis involved a multiple linear regression equation.

**Conclusions:**

The average amount of bone available for use in coracoid transfer in Mongolian men was less than that of other populations. Mutation of the pectoralis minor may induce intraoperative capsule injury because this muscle passes over the coracoid deep to the joint capsule of the glenohumeral joint and constitutes part of the shoulder joint, strengthening the joint. Statistically, higher coracoids appeared in shorter patients and longer coracoids appeared in taller patients. Surgically, great care should be taken to consider a patient’s height to precisely implement the congruent-arc Latarjet technique.

## Background

Anterior shoulder instability is a common sports-related injury. In the past 15 years, available surgical options for arthroscopic treatment of anterior shoulder instability have increased considerably. However, the failure rate remains high, especially in patients with significant glenohumeral bone defects [1]. In patients with glenoid bone loss, glenoid bone augmentation using arthroscopic coracoid transfer is helpful in reducing dislocation rates [2–4]; however, coracoid process anatomy varies by region. In 2011, Dolan et al. [5] reported that the mean maximum length of the coracoid available for transfer was 28.5 mm. In 2012, Terra et al. [6] stated that the safety margin for osteotomy was 26.4 mm. A search of the most recent 20 years of the Chinese Medical Database revealed only one article on coracoid anatomy, and the study used dehydrated scapulae, which contained no soft tissue. The aims of our study were to describe the soft tissue attachments on the coracoid as they relate to the bony anatomy, define the average amount of bone available for use in coracoid transfer, analyze the characteristics of the pectoralis minor and coracoid, and study the relationship between the bony dimensions of the coracoid and body length in Mongolian men.

## Methods

We used 30 fresh-frozen shoulders from 15 deceased donors (all Mongolian men) with an average age of 60.8 years (range, 40–71 years) and an average body length, defined as the distance from the medial malleolus to the ipsilateral arcus superciliaris, of 149.6 cm (range, 140.0–167.0 cm). The skin, subcutaneous tissue, deltoid, and overlying soft tissue were removed, exposing the coracoid, lateral clavicle, and acromion. The footprints of each ligament and tendon attached to the coracoid were preserved. The footprint insertion areas were clearly marked circumferentially with a pen while the ligament/tendon footprint was still intact. The ligament/tendon was then incised, leaving a short stump.

Measurements were independently recorded by two researchers blinded to each other’s measurements, using a digital caliper (Mitutoyo America Corp., Aurora, IL) accurate to 0.2 mm. Measurements from the two researchers were then averaged and recorded. The following bony dimensions of the coracoid were measured: (1) distance from the coracoid tip to the coracoid base (length), (2) coracoid tip width, (3) coracoid tip height, (4) distance from the coracoid tip to the coracoid midpoint (hereafter termed “midpoint”), (5) midpoint width, and (6) midpoint height (Figs. 3 and 4).

**Fig. 1:**
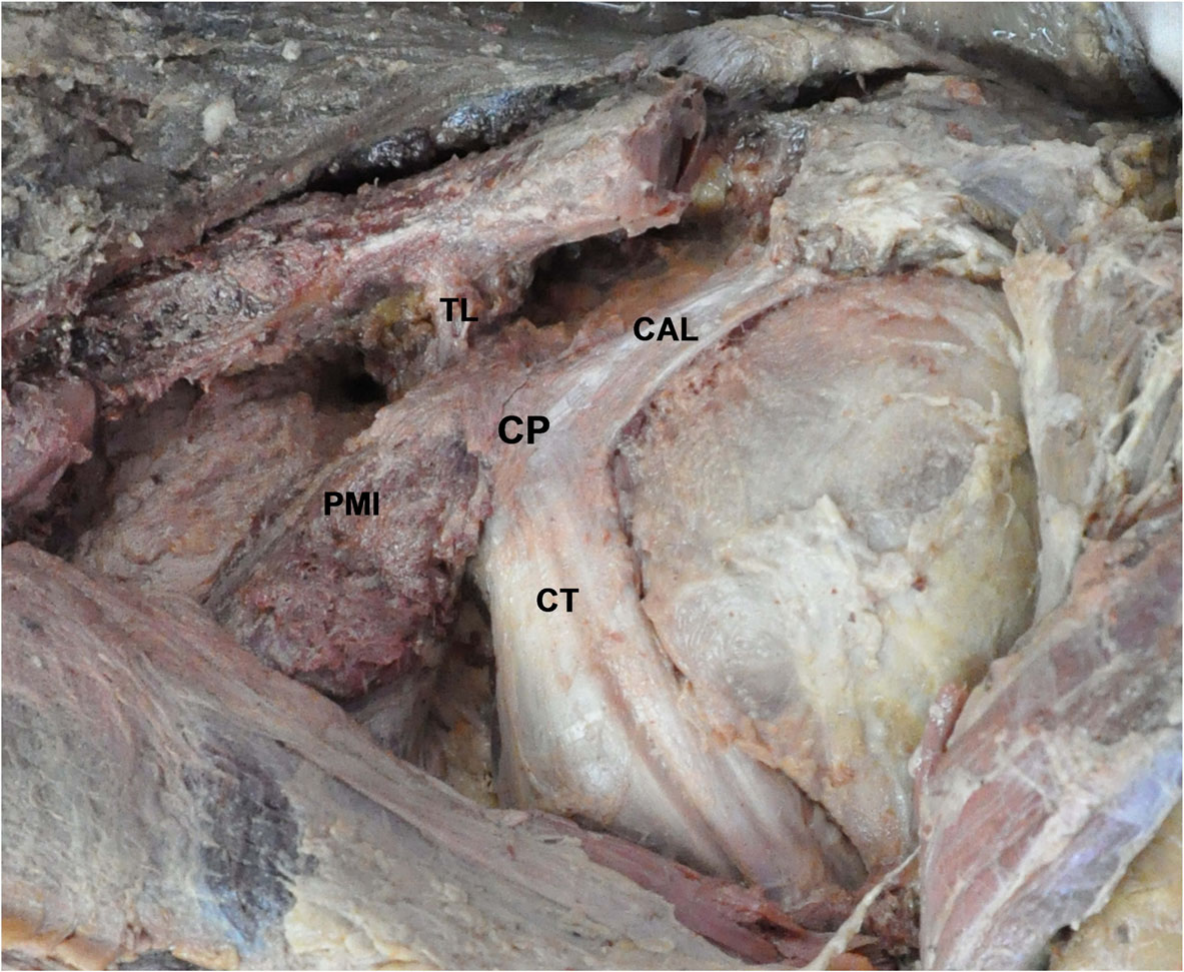
Anatomical structures in an isolated coracoid process of the left shoulder. *CAL* coracoacromial ligament, *CP* coracoid process, *CT* conjoint tendon, *PMI* pectoralis minor, *TL* trapezoid ligament

**Fig. 2:**
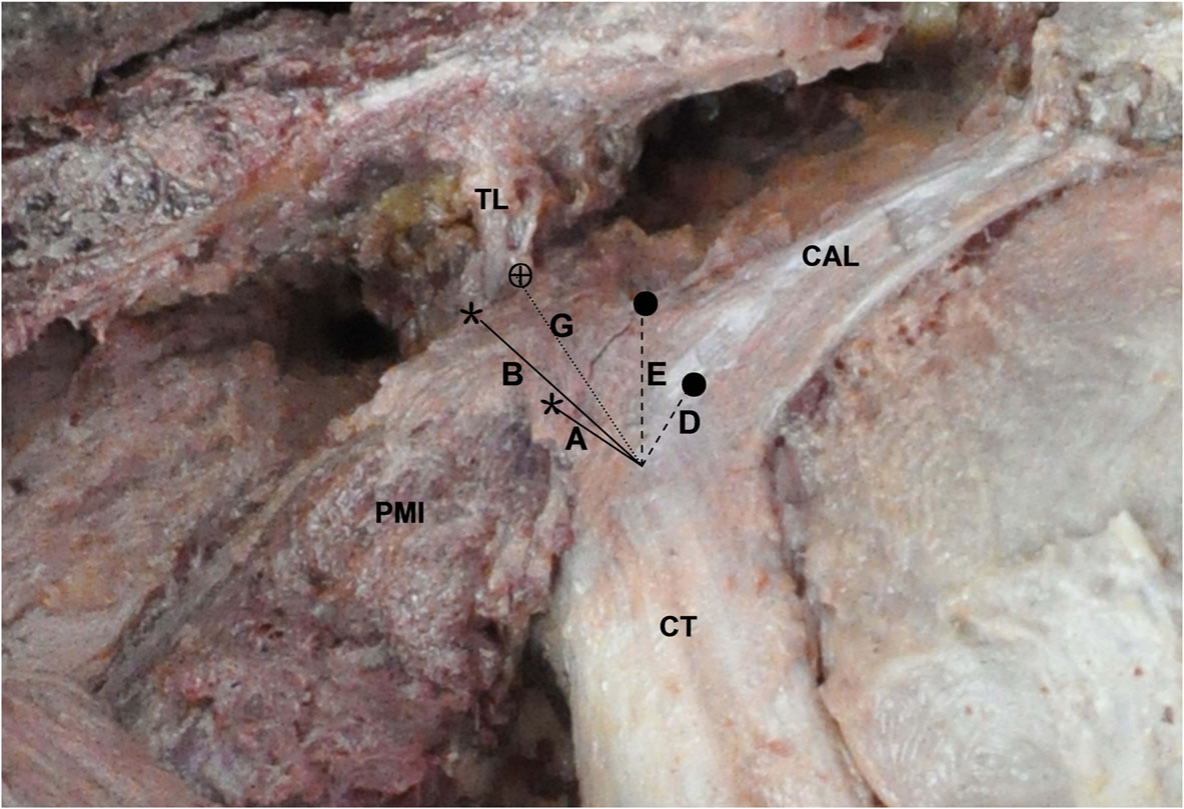
Anatomical structures of the coracoid process of the left shoulder. The *numbers* correspond to Table 1. *A*, coracoid tip to anterior pectoralis minor; *B*, coracoid tip to posterior pectoralis minor; *D*, coracoid tip to anterior coracoacromial ligament; *E*, coracoid tip to posterior coracoacromial ligament; *G*, coracoid tip to anterior coracoclavicular (C–C) trapezoid ligament (safety margin); *asterisk*, anterior and posterior margins of pectoralis minor; *filled circle*, anterior and posterior margins of coracoacromial ligament. *CAL* coracoacromial ligament, *PMI* pectoralis minor, *TL* trapezoid ligament, *CT* conjoint tendon

The following soft tissue footprints on the coracoid were also studied: pectoralis minor, coracoacromial ligament, trapezoid ligament, conjoint tendon, and coracoid process (Fig. 1). The distance between these soft tissue footprints and their distances from the measured bony landmarks were also measured (Fig. 2). The distance from the coracoid tip to the anterior trapezoid ligament was marked “G” (safety margin, Fig. 5). This bony area anterior to the trapezoid ligament is often described as the “knee” or “elbow” of the coracoid. In this study, the trapezoid footprint was located more anteriorly on the coracoid; therefore, the trapezoid ligament was considered more clinically relevant than the coracoid ligament in the osteotomy and transfer of the coracoid. The distance from the medial malleolus to the ipsilateral superciliary arches was also measured, because although the skull and calcanei had been dissected, the medial malleolus and superciliary arches remained intact. All data were statistically analyzed using SPSS ver. 15.0 (SPSS Inc., Chicago, IL).

## Results

**Fig. 3:**
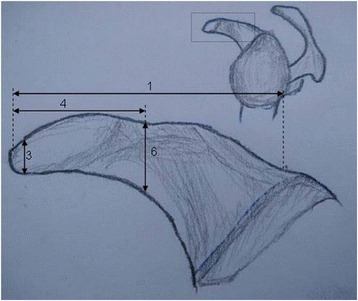
View of the lateral aspect of the coracoid. The *numbers* correspond to Table 1. *1*, coracoid length (distance from coracoid tip to base); *3*, coracoid tip height; *4*, distance from the coracoid tip to the coracoid midpoint; *6*, midpoint height

**Fig. 4:**
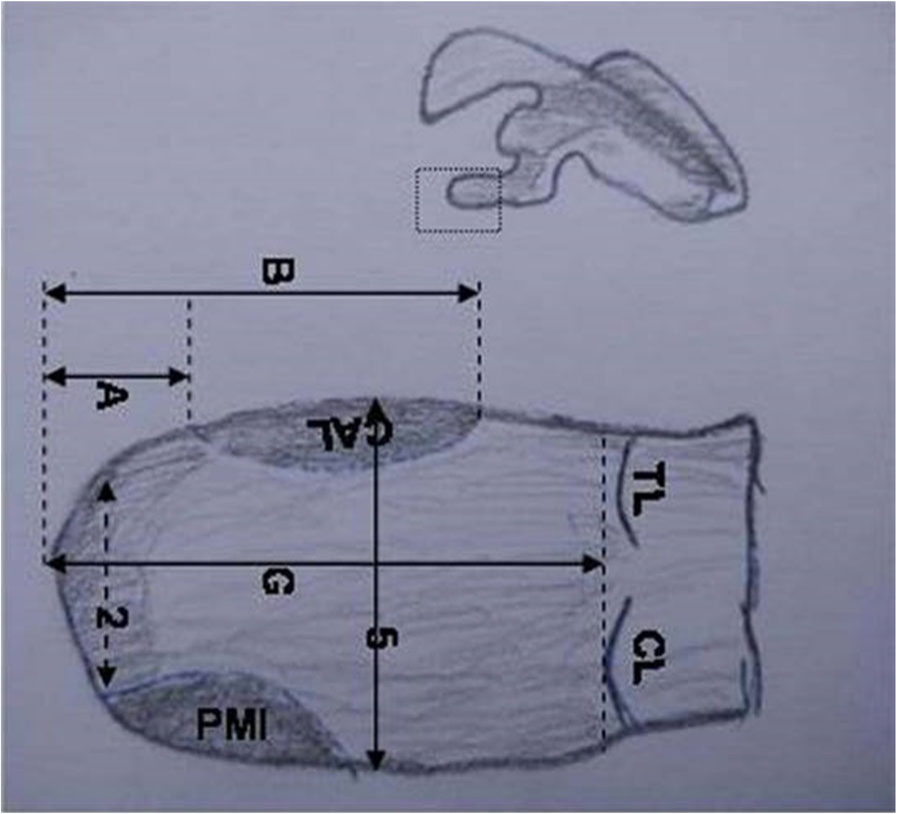
View of superior aspect of the coracoid. The *numbers* correspond to Table 1. *2*, coracoid tip width; *5*, midpoint width; *A*, coracoid tip to anterior pectoralis minor; *B*, coracoid tip to posterior pectoralis minor; *G*, coracoid tip to anterior coracoclavicular (C–C) trapezoid ligament. *CAL* coracoacromial ligament, *PMI* pectoralis minor, *TL* trapezoid ligament, *CL* coracoid ligament

**Fig. 5:**
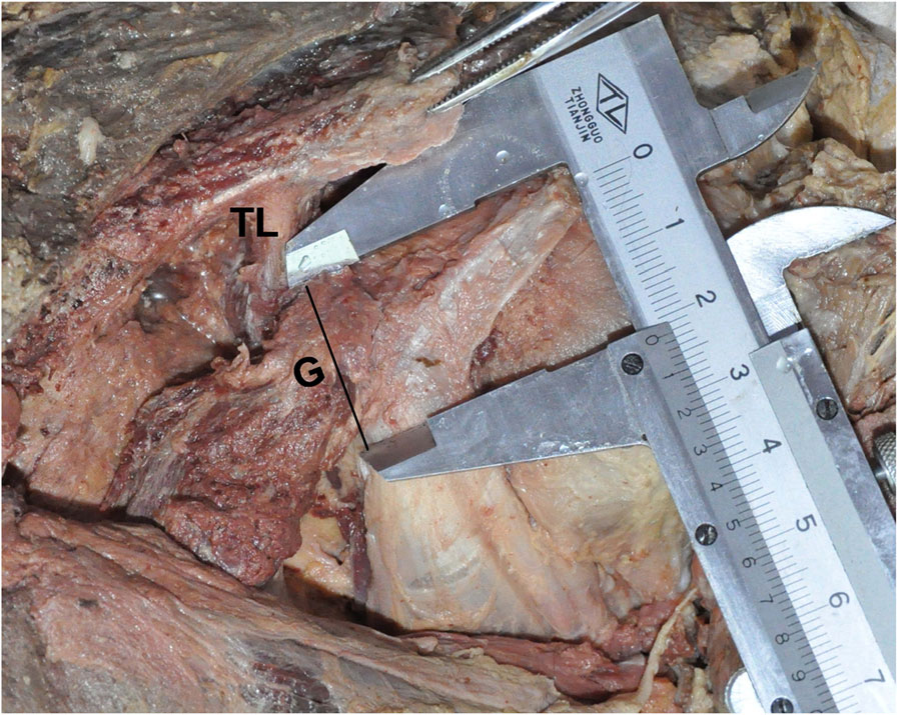
Anatomical structures of the coracoid process of the left shoulder. Trapezoid ligament (*TL*); *G*, coracoid tip to anterior coracoclavicular (C–C) trapezoid ligament (safety margin)

The anatomical dimensions of the coracoid bony anatomy are provided in Table 1 and Figs. 1, 2, 3, 4, and 5. The mean coracoid length (1) was 42.10 ± 2.3 mm. The mean coracoid tip width (2) was 13.61 ± 2.00 mm, and the mean coracoid tip height (3) was 9.10 ± 1.75 mm. The maximum length of the coracoid available for transfer (i.e., the bone segment between the tip of the coracoid and the anterior extent of the trapezoid coracoclavicular ligament or tip to “knee” or “elbow” of the coracoid) (G) was 23.93 ± 2.32 mm. The midpoint of the coracoid (4) was 24.75 ± 7.23 mm from the tip. The mean width of the midpoint (5) was 15.29 ± 1.70 mm, and the mean height of the midpoint (6) was 11.61 ± 1.98 mm. The mean distance from the coracoid tip to the anterior pectoralis minor (A) was 8.53 ± 1.78 mm and to the posterior pectoralis minor (B) was 19.67 ± 1.89 mm. The mean anteroposterior width of the pectoralis minor footprint (C) was 12.76 ± 1.62 mm. The mean distance from the coracoid tip to the anterior coracoacromial ligament (D) was 9.67 ± 2.96 mm and to the posterior coracoacromial ligament (E) was 18.75 ± 5.46 mm. The anteroposterior width of the coracoacromial ligament footprint (F) was 13.93 ± 4.82 mm.

**Table 1:** Bony anatomy dimensions of the coracoid and relationships between the coracoid soft tissue footprints

Variable	Mean (mm)	SD (mm)	Minimum (mm)	Maximum (mm)	IN (99 %)^a^	
					Minimum	Maximum
1 Coracoid length	42.1	2.31	38.26	46.66	40.7	43.49
2 Tip width	13.61	2	10.65	16.93	12.41	14.82
3 Tip height	9.1	1.75	6.91	12.37	8.04	10.16
4 Distance from tip to midpoint	24.75	7.23	12.42	33.04	20.38	29.13
5 Midpoint width	15.29	1.7	12.54	19.26	14.26	16.32
6 Midpoint height	11.61	1.98	8.73	14.27	10.42	12.81
A Distance from tip to anterior pectoralis minor	8.53	1.78	5.97	12.21	7.45	9.6
B Distance from tip to posterior pectoralis minor	19.67	1.89	16.48	22.98	18.53	20.82
Pectoralis minor insertion AP width	12.76	1.62	9.97	14.99	11.78	13.73
D Coracoid tip to anterior coracoacromial ligament	9.67	2.96	6.57	15.2	7.88	11.45
E Coracoid tip to posterior coracoacromial ligament	18.75	5.46	12.17	31.42	15.45	22.05
Coracoacromial ligament insertion AP width	13.93	4.82	9.02	24.22	11.02	16.84
G Coracoid tip to anterior coracoclavicular trapezoid ligament (safety margin)	23.93	2.32	20.65	29.31	22.53	25.33

^a^Descriptive measurements with 99 % of normality interval

**Fig. 6:**
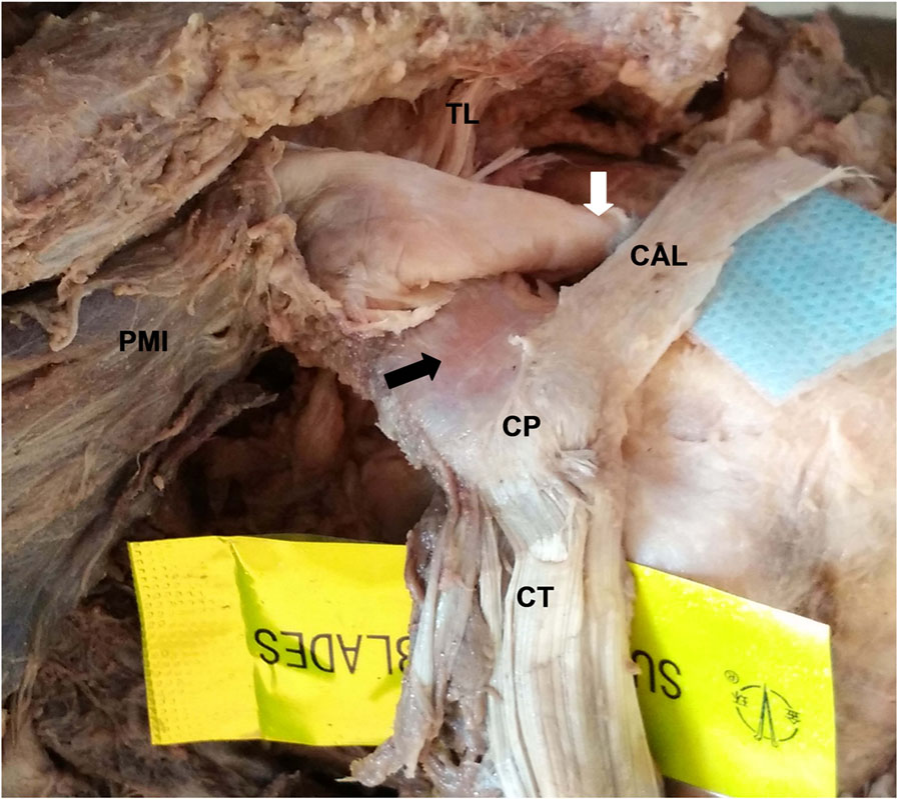
Anatomical structures associated with the isolated coracoid process of the left shoulder with emphasis on the variation. *Black arrow* indicates the coracoid acting as a lever for the pectoralis minor as the muscle passes over it. *White arrow* indicates the ligament of the pectoralis minor, deep to the joint capsule of the glenohumeral joint. *CAL* coracoacromial ligament, *CP* coracoid process, *CT* conjoint tendon, *PMI* pectoralis minor, *TL* trapezoid ligament

The pectoralis minor was part of the joint capsule and passed over the coracoid in some samples. Statistically, the mutation rate of the footprint of the pectoralis minor, which was asymmetrical and irregular, was 23.33 % (Fig. 6, Table 2). Statistical analysis involved the following multiple linear regression equation: *Y* = −16.747 + 4.971*X*_1_ − 3.469*X*_2_ − 0.536*X*_3_, where *Y* = the distance from the medial malleolus to the ipsilateral superciliary arches, *X*_1_ = the distance from the coracoid tip to the anterior border of the coracoclavicular ligament, *X*_2_ = the height of the coracoid midpoint, and *X*_3_ = the height of the coracoid tip.

**Table 2:** Distance from medial malleolus to ipsilateral arcus superciliaris and mutation of the pectoralis minor footprint

Variable	Cadaver 1		Cadaver 2		Cadaver 3		Cadaver 4		Cadaver 5		Cadaver 6		Cadaver 7		Cadaver 8		Cadaver 9		Cadaver 10		Cadaver 11		Cadaver 12		Cadaver 13		Cadaver 14		Cadaver 15	
	L	R	L	R	L	R	L	R	L	R	L	R	L	R	L	R	L	R	L	R	L	R	L	R	L	R	L	R	L	R
Distance from the malleolus to the ipsilateral arcus superciliaris (CM)	148.0		158.5		147.0		147.0		152.0		155.0		140.0		143.0		144.0		136.0		167.0		156.0		149.0		155.0		146.0	
Mutation of the footprint of the pectoralis minor	−	−	+	+	−	−	−	−	−	−	−	−	+	+	−	−	−	−	−	−	+	−	−	−	−	+	−	−	−	+

*L* left shoulder, *R* right shoulder; +, mutation of the footprint of the pectoralis minor; −, no mutation of the footprint of the pectoralis minor

## Discussion

In 1954, Latarjet [7] described an osteotomy where the reference was the base of the coracoid process. In 1958, Helfet [8] described the Bristow procedure where the cut was made 10 mm from the coracoid tip with the insertion of the conjoint tendon. May [9] modified the technique, advocating a cut just proximal to the insertion of the coracobrachialis tendon. Lafosse et al. [10] inserted a 20–25-mm bone fragment into the glenoid together with a portion of the coracoacromial ligament and tendon. Burkhart et al. [11] described an osteotomy distal to the angle (elbow) of the coracoid process; however, the exact location was unclear and may have been the angle or elbow of the coracoid process.

Currently, most surgeons comply with the “safety margin” principle and avoid the coracoclavicular ligaments; however, the required safe distance is not well defined. In 2011, Dolan et al. [5] reported the safety margin as 28.5 mm. In 2012, Terra et al. [6] described the safety margin for osteotomy as 26.4 mm and another study recommended that the coracoid bone block should be approximately 20.0 mm in length [12]. In our study, a safety margin of 23.93 ± 2.32 mm was derived from the male Mongolian donors, and no coracoid abnormalities for obstetric brachial plexus injury in children [13], which differ slightly from the two previous studies. This result may be attributed to differences in race, but further study is required to confirm this conclusion.

Rockwood et al. [14] reported that the mutation rate of the footprint of the pectoralis minor was 15 %; however, in some cadaver shoulders in our study, the pectoralis minor passed from the anterior surfaces of ribs III–V and also passed over the coracoid process deep to the joint capsule of the glenohumeral joint as part of the shoulder joint capsule. The mutation rate in our study was 23.33 %, and the anatomy was asymmetrical and irregular. These findings indicate that when operating using the Latarjet procedure, surgeons should carefully consider the possibility of a pectoralis minor malformation to decrease the risk of iatrogenic injury. If the coracoid lever is destroyed during surgery, the pectoralis minor is loosened, which injures the superior aspects of the glenohumeral joint, unbalancing the shoulder. A limitation in our study is the small sample size, which may have introduced an error. Future studies with larger sample sizes are needed to validate our findings.

In 2009, De Beer and Roberts modified the classic Latarjet technique and named it the congruent-arc Latarjet technique [15]. This technique involves rotating the coracoid graft by 90° along its longitudinal axis and transferring it such that the inferior surface reconstitutes the glenoid articulation, then fixing it with two 3.5-mm screws. Hantes et al. [16] illustrated the ability of the congruent-arc Latarjet procedure to treat large glenoid defects. However, when the coracoid thickness (8.4–11 mm or ~60 % of its width) is considered, it becomes apparent that the congruent-arc procedure produces a cantilevered geometry with a smaller contact area with the native glenoid, which may predispose to graft fixation issues. Our results showed that the coracoid width was longer than its height, similar to the conclusions of Degen et al. [17]. Nevertheless, based on multiple linear regression analysis, we concluded that the safety margin was proportional to the cadaver’s height, and the coracoid tip and midpoint height were inversely proportional to the cadaver’s height. Clinically, these relationships mean that taller patients have shorter coracoid height but longer coracoid length. Therefore, great care should be taken intraoperatively to consider a patient’s height to precisely implement the congruent-arc Latarjet technique. For example, congruent-arc Latarjet is not an option in a taller patient with shorter coracoid height because there is inadequate space for two 3.5-mm screws. The Latarjet procedure is the best choice in patients with longer coracoids.

There is no single criterion to determine which patient would benefit from the Latarjet procedure vs. the congruent-arc procedure. Boons et al. [18] stated that the classic Latarjet and congruent-arc techniques do not result in differences in the range of motion and stiffness of the shoulder. However, the congruent-arc Latarjet technique is useful in placing the humeral head forward in the glenohumeral joint. Currently, neither method is considered clearly superior to the other.

A limitation of this study involves the methodology of studying a curved three-dimensional anatomical structure. Consistently establishing the longest axis and the most anterior portion or tip of the coracoid process is challenging because of its curved and tortuous morphology. Another limitation was that, as a cadaver anatomical study, no detailed history could be obtained, and no women were included.

## Conclusions

The purpose of this study was to describe the coracoid bony anatomy and its soft tissue insertions in Mongolian men to assist surgeons during preoperative planning and decision-making for shoulder surgery. Our results established a safety margin of 23.93 ± 2.32 mm for osteotomy in Mongolian men. We recommend that careful attention to the mutation rate of the pectoralis minor may influence operative results. Statistically, in our study, higher coracoids were present in shorter patients; therefore, clinically, we recommend that surgeons consider a patient’s height carefully to precisely implement the modified Latarjet techniques.

## References

[1] Lewington MR, Urquhart N, Wong IH (2015). Lateral decubitus all-arthroscopic Latarjet procedure for treatment of shoulder instability. Arthrosc Tech.

[2] Yamamoto N, Muraki T, An KN, Sperling JW, Cofield RH, Ltoi E (2013). The stabilizing mechanism of the Latarjet procedure: a cadaveric study. J Bone Joint Surg Am.

[3] Giles JW, Degen RM, Johnson JA, Athwal GS (2014). The Bristow and Latarjet procedures: why these techniques should not be considered synonymous. J Bone Joint Surg Am.

[4] Dumont GD, Fogerty S, Rosso C, Lafosse L (2014). The arthroscopic Latarjet procedure for anterior shoulder instability: 5-year minimum follow-up. Am J Sports Med.

[5] Dolan CM, Hariri S, Hart ND, McAdams TR (2011). An anatomic study of the coracoid process as it relates to bone transfer procedures. J Shoulder Elbow Surg.

[6] Terra BB, Ejnisman B, de Figueiredo EA, Cohen C, Monteiro GC, de Castro PA (2013). Anatomic study of the coracoid process: safety margin and practical implications. Arthroscopy.

[7] Latarjet M (1954). Treatment of recurrent dislocation of the shoulder. Lyon Chir.

[8] Helfet AJ (1958). Coracoid transplantation for recurring dislocation of the shoulder. J Bone Joint Surg Br.

[9] May VR (1970). A modified Bristow operation for anterior recurrent dislocation of the shoulder. J Bone Joint Surg Am.

[10] Lafosse L, Lejeune E, Bouchard A, Kakuda C, Gobezie R, Kochhar T (2007). The arthroscopic Latarjet procedure for the treatment of anterior shoulder instability. Arthroscopy.

[11] Burkhart SS, De Beer JF, Barth JR, Cresswell T, Roberts C, Richards DP (2007). Results of modified Latarjet reconstruction in patients with anteroinferior instability and significant bone loss. Arthroscopy.

[12] Aurich M, Hofmann GO, Gras F (2015). Reconstruction of the coracoacromial ligament during a modified Latarjet procedure: a case series. BMC Musculoskelet Disord.

[13] Nath RK, Mahmooduddin F, Liu X, Wentz MJ, Humphries AD (2010). Coracoid abnormalities and their relationship with glenohumeral deformities in children with obstetric brachial plexus injury. BMC Musculoskelet Disord.

[14] Rockwood, Masten, Wirth, Lippitt. The shoulder. 4th ed. Elsevier; 2009.

[15] De Beer JF, Roberts C (2010). Glenoid bone defects-open Latarjet with congruent arc modification. Orthop Clin North Am.

[16] Hantes ME, Venouziou A, Bargiotas KA, Metafratzi Z, Karantanas A, Malizos KN (2010). Repair of an anteroinferior glenoid defect by the Latarjet procedure: quantitative assessment of the repair by computed tomography. Arthroscopy.

[17] Degen RM, Giles JW, Boons HW, Litchfield RB, Johnson JA, Athwal GS (2013). A biomechanical assessment of superior shoulder translation after reconstruction of anterior glenoid bone defects: the Latarjet procedure versus allograft reconstruction. Int J Shoulder Surg.

[18] Boons HW, Giles JW, Elkinson I, Johnson JA, Athwal GS (2013). Classic versus congruent coracoid positioning during the Latarjet procedure: an in vitro biomechanical comparison. Arthroscopy.

