# Erratum to: Anatomical study of the coracoid process in Mongolian male cadavers using the Latarjet procedure

**DOI:** 10.1186/s13018-017-0538-7

**Published:** 2017-03-13

**Authors:** Jianqiang Lian, Lele Dong, Yanjun Zhao, Jinlei Sun, Wenlong Zhang, Chunzheng Gao

**Affiliations:** 1 The First Affiliated Hospital of Baotou Medical College, Baotou, Inner Mongolia China; 2 The Second Hospital of Shandong University, Jinan, Shandong China

## Erratum

The author affiliation of one author in the original publication [1] contains an incorrect unit. The correct unit is the following: “The Second Hospital of Shandong University, Jinan, Shandong, China.”
